# Synthesis of 2-methoxy benzoquinone and 2,6-dimethoxybenzoquinone by selected lactic acid bacteria during sourdough fermentation of wheat germ

**DOI:** 10.1186/1475-2859-12-105

**Published:** 2013-11-11

**Authors:** Carlo Giuseppe Rizzello, Thomas Mueller, Rossana Coda, Franziska Reipsch, Luana Nionelli, José Antonio Curiel, Marco Gobbetti

**Affiliations:** 1 Dipartimento di Scienze del Suolo, della Pianta e degli Alimenti, University of Bari, 70126 Bari, Italy; 2 Department of Internal Medicine IV, Oncology/Hematology, Martin-Luther-University Halle-Wittenberg, Halle/Saale, Germany

**Keywords:** Wheat germ, Lactic acid bacteria, Cancer, Quinones, β-glucosidase activity

## Abstract

**Background:**

In the last decade, several studies described the promising cytotoxic activity of fermented wheat germ towards cancer cell lines and during *in vivo* clinical trials. Recent data suggested that the antiproliferative, antimetastatic and immunological effects of this preparation are mainly attributed to quinones. This study aimed at exploiting the potential of sourdough lactic acid bacteria fermentation to release 2-methoxy benzoquinone, and 2,6-dimethoxybenzoquinone, which are naturally present in wheat germ as glycosylated and non-physiologically active form.

**Results:**

Preliminarily, forty strains of lactic acid bacteria, previously isolated from wheat germ, were in vitro screened based on β-glucosidase activity. *Lactobacillus plantarum* LB1 and *Lactobacillus rossiae* LB5 were selected based on the highest enzyme activity and on technology features. These strains were used in combination to ferment wheat germ. Raw wheat germ, without bacterial inoculum, was subjected to the same incubation and used as the control. The sourdough fermented wheat germ was characterized based on microbiological, physico-chemical and biochemical features. During incubation, the release of the non-glycosylated and physiologically active 2-methoxy benzoquinone, and 2,6-dimethoxybenzoquinone was almost completed during 24 h. Compared to the control, the concentration of the above bioactive compounds increased almost 4 and 6-folds. Both raw wheat germ (control) and sourdough fermented wheat germ were *ex vivo* assayed for the anti-proliferative activity towards various cell lines of germ cell tumor, colon carcinoma and ovarian carcinoma. While no effect was found for the raw wheat germ, the sourdough fermented preparation markedly and variously affected the human tumor cell lines. The values of IC_50_ ranged from 0.105 ± 0.005 to 0.556 ± 0.071 mg/ml, with a median value of IC_50_ of 0.302 mg/ml.

**Conclusions:**

These results are comparable to those found for other well-known pharmaceutical preparations, and may disclose the use of the sourdough fermented wheat germ as an ingredient, nutritional supplement and/or anticancer drug.

## Background

Wheat germ, corresponding to 2-3% of the total weight of wheat kernel, is almost systematically removed during milling since it adversely affects the shelf-life and the processing quality of the flour [1]. Due to the high concentration of functional compounds, wheat germ is one of the most attractive and promising source of α-tocopherol, vitamins of group B, dietary fiber, polyunsatured fats, minerals and phytochemicals [1]. Usually, the effects of wheat bran on human physiology are grouped into nutritional, mechanical (mainly due to the fiber content) and antioxidant (mainly due to phenolic acid and alkylresorcinols) [2].

Besides the positive effects, the use of wheat germ in bakery industry is still challenging because of the poor stability and the presence of anti-nutritional factors such as: (i) raffinose which is not digested by pancreatic enzymes but metabolized by gas-producing bacteria of the large intestine, thus causing disorders such as flatulence [1]; (ii) phytic acid which markedly decreases the mineral bioavailability [3]; and (iii) wheat germ agglutinin (WGA) which is responsible for the hyperplastic and hypertrophic growth of the small bowel and pancreas [4]. Treatments by heat, microwave and extrusion [4] or the addition of antioxidants [5] were considered to increase stability of wheat germ. Despite their effectiveness, these technology approaches are in some cases expensive, not completely resolving, and somewhat decreasing the nutritional value of wheat germ [5]. Recently, some studies exploited the potential of microbial enzymes or sourdough fermentation to process wheat germ and to enhance the nutritional and sensory properties of related cereal based foods [1]. Lactic acid bacteria isolated from wheat germ were selected based on technology features and used as starters for the manufacture of fermented wheat germ through a biotechnology, which resembled the traditional sourdough fermentation [1]. Sourdough fermentation is one of the oldest food biotechnologies, which was extensively studied for the effects on the sensory, structural, nutritional and shelf life properties of leavened baked goods [6]. Acidification, proteolysis and activation of a number of enzymes as well as the synthesis of microbial metabolites cause several changes during sourdough fermentation, which affect the dough and baked good matrix and, in turn, influence the nutritional/functional quality [6]. Overall, sourdough fermentation stabilized and enhanced the nutritional properties of wheat germ. Besides, lactic acidification markedly decreased the lipase activity of the sourdough fermented wheat germ compared to the levels found in the raw wheat germ [1].

In the last decade, several studies described the promising cytotoxic activity of a yeast (*Saccharomyces cerevisiae)* fermented wheat germ extract (Avemar®) towards cancer cell lines and during *in vivo* clinical trials [7,8]. Recent data suggested that the antiproliferative, antimetastatic and immunological effects of this preparation are mainly attributed to two quinones, 2-methoxy benzoquinone (2-MBQ), and 2,6-dimethoxybenzoquinone (2,6-DMBQ) [7].

This study aimed at investigating the effect of the sourdough lactic acid bacteria fermentation on the release of 2-methoxy benzoquinone, and 2,6-dimethoxybenzoquinone, which are naturally present in the wheat germ as glycosylated and non-physiologically active form. The cytotoxic activity of the sourdough fermented wheat germ was determined through *ex vivo* assays on several human cancer cell lines.

## Results

### β-glucosidase activity

**Figure 1:**
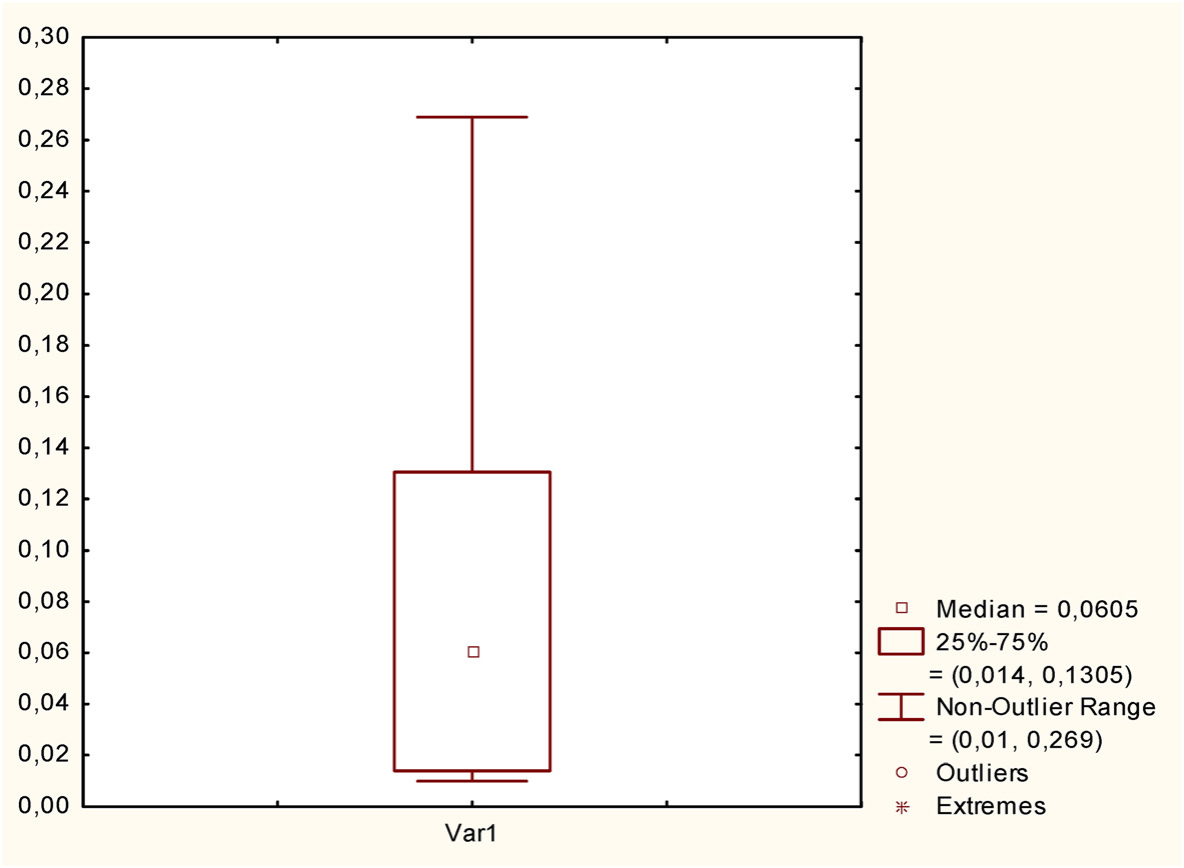
**β-glucosidase activity.** β-glucosidase activity of lactic acid bacteria strains isolated from wheat germ, measured in terms of *p*-nitrophenol released from *p*-nitrophenyl-β-D-glucopyranoside (*p*NPG) substrate according to the protocol described by De Angelis et al. [10]. One unit (U) of enzyme activity was defined as the amount of *β*-glucosidase that released 1 μmol of *p*-nitrophenol from the substrate *p*NPG per milliliter per min under the assay conditions. The aggregate data of the strains are shown in a box-plot. The center line of the box represents the median (□), the top and bottom of the box represent the 25th and 75th percentile of the data, respectively. The top and bottom of the bars represent the 5th and 95th percentile of the data, respectively.

Preliminarily, the β-glucosidase activity of the forty strains of lactic acid bacteria, previously isolated from wheat germ, was assayed on the synthetic substrate *p*NPG. Figure 1 summarizes the results. The enzyme activity varied within a wide range: from 0.011 ± 0.005 to 0.269 ± 0.014 U. The median value of the distribution was found to be 0.06 U, and the values corresponding to the 25^th^ and 75^th^ percentile of the data were 0.014 and 0.130 U, respectively. In particular, the well-characterized strains [1,9]*Lactobacillus plantarum* LB1 and *Lactobacillus rossiae* LB5 showed the highest β-glucosidase activity (0.269 ± 0.014 U) and a value located in the non-outlier range (higher than the 75^th^ percentile of the data, 0.140 ± 0.008 U), respectively. Besides *L. plantarum* LB1 and *L. rossiae* LB5, *Weissella confusa* G8 (0.062 ± 0.005 U), and *Pediococcus pentosaceus* G1 (0.015 ± 0.004 U), representative of the strains having β-glucosidase activity close to the median value, and of the strains having low enzymatic activity, respectively, were used for wheat germ fermentation with the aim of investigating the correlation with the benzoquinones release. Nevertheless, based on this preliminary assay and since both *L. plantarum* LB1 and *L. rossiae* LB5 were previously selected based on the kinetic of acidification and other technology features [1,9], these two strains were further used in combination to ferment wheat germ.

### Sourdough fermentation of wheat germ

The averaged values of raw wheat germ (RWG) were as follows: moisture 11.08 ± 0.22%, protein (N x 5.70) 28.50 ± 0.72% of dry matter (d.m.); fat 7.95 ± 0.06% of d.m.; and ash 3.82 ± 0.09% of d.m. RWG had pH of 6.36 ± 0.05 and Total titratable acidity (TTA) of 18.3 ± 0.11 ml of 0.1 M NaOH/10 g. The concentration of total free amino acids was 15614 ± 134 mg/kg. As estimated by plate count, cell density for yeasts and presumptive lactic acid bacteria in RWG was 1.5 ± 0.5 x 10^3^ and 2.0 ± 0.5 x 10^5^ cfu/g, respectively. After 24 h of incubation at 30°C, the cell density increased to 5.5 ± 1.5 x 10^4^ and 4.2 ± 0.7 x 10^7^ cfu/g, respectively, and the pH decreased to 5.51 ± 0.07.

The persistence and the dominance of *L. plantarum* LB1 and *L. rossiae* LB5 during fermentation of SFWG was confirmed through RAPD-PCR analysis. As estimated by plate count and the use of RAPD-PCR, cell densities at the end of fermentation were 6.5 ± 0.4 x 10^9^ and 2.3 ± 0.5 x 10^9^ cfu/g for *L. plantarum* LB1 and *L. rossiae* LB5, respectively. The pH of the sourdough fermented wheat germ (SFWG) was 4.16 ± 0.03. Compared to RWG, TTA increased to 24.9 ± 0.07 ml of 0.1 M NaOH/10 g.

SFWG was further characterized for features previously recognized as related to nutritional and technology quality [1,9]. The water/salt-soluble extracts of RWG and SFWG were used to determine the lipase activity. The minimum concentration of the crude enzyme extract that failed to give a detectable zone of hydrolysis was, respectively, 50.2 ± 1.4 and 163.8 ± 1.6 μg/ml. After fermentation, the concentration of total free amino acids increased to 23491 ± 94 mg/kg. Almost all the free amino acids increased. Leu, Lys, Phe, Val, His, Ala, and Met showed the highest increase, which varied from 2 (Ala) to ca 10-fold (Leu and Met) compared to the levels found in RWG. Apart from the increase during fermentation, Arg, Ser, GABA and Lys were the free amino acids found at highest concentration in SFWG (4070 ± 21, 2252 ± 14, 2031 ± 18 and 1944 ± 27 mg/kg, respectively). Lactic and acetic acids were not detectable in RWG. After fermentation, 0.98 ± 0.02 and 0.25 ± 0.02% of lactic and acetic acids were, respectively, found in SFWG.

The methanol extracts of RWG and SFWG contained 0.49 ± 0.02 and 0.65 ± 0.02 mM of total phenols (expressed as gallic acid equivalent), respectively.

### Release of 2-methoxy benzoquinone and 2,6-dimethoxybenzoquinone during sourdough fermentation

As determined by selective extraction and HPLC analysis, RWG contained 2-methoxy benzoquinone and 2,6-dimethoxybenzoquinone at the concentrations of 0.082 ± 0.01 and 0.035 ± 0.005 mg/g, respectively. After sourdough fermentation with *L. plantarum* LB1 and *L. rossiae* LB5 [1], the concentrations of the above two quinones increased to 0.415 ± 0.016 and 0.252 ± 0.013 mg/g, respectively. Compared to SFWG obtained by the inoculum of the two starters together, the concentration of both benzoquinones in wheat germ fermented with the lactic acid bacteria singly was ca. 70, 40, 10%, and not detectable for *L. plantarum* LB1, *L. rossiae* LB5, *W. confusa* G8, and *P. pentosaceus* G1, respectively.

**Figure 2:**
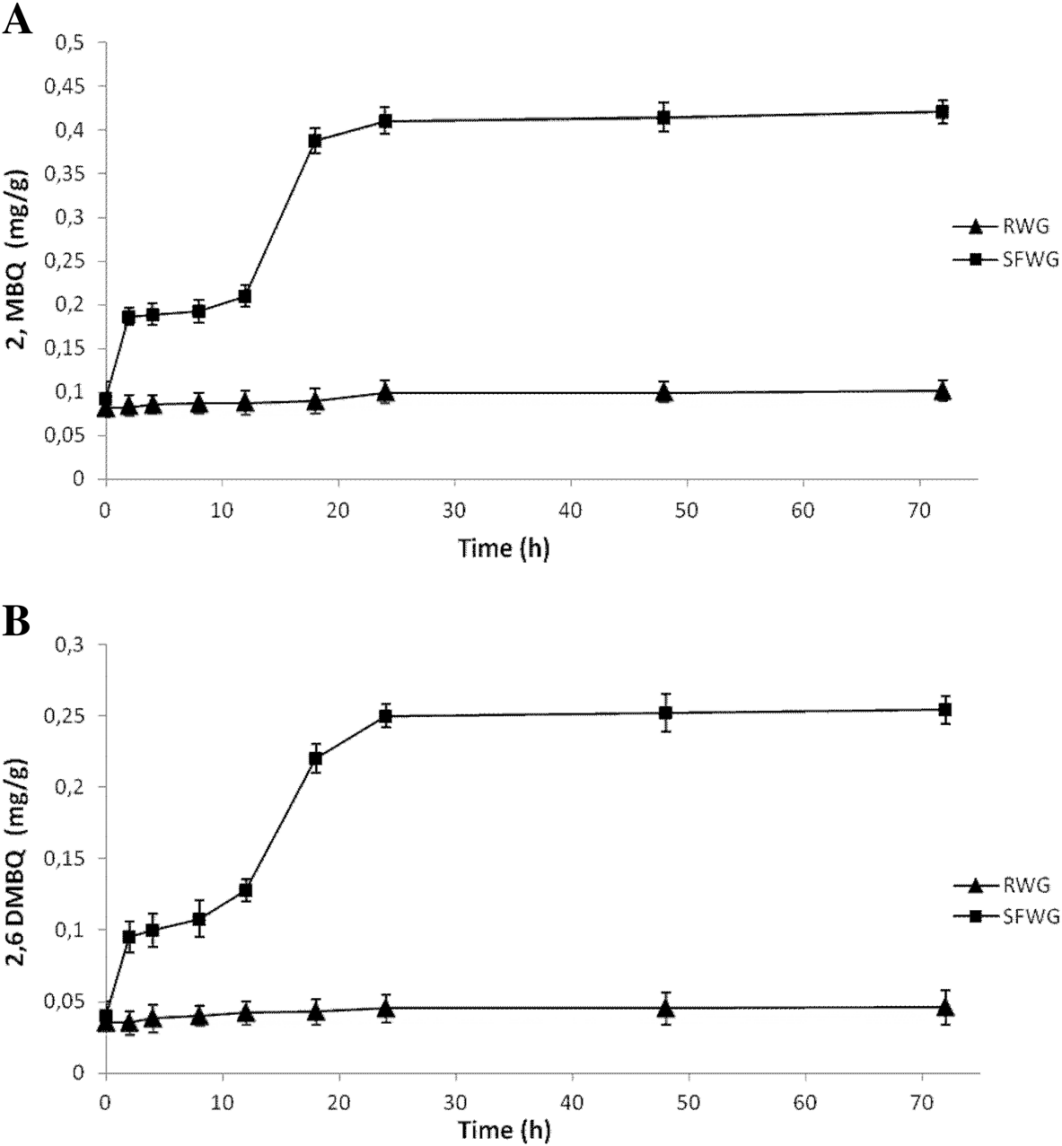
**Release of quinones during fermentation.** Concentrations (mg/g) of 2-methoxy benzoquinone (2,MBQ) **(panel A)** and 2,6-dimethoxybenzoquinone (2,6 DMBQ) **(panel B)** (mg/g) of sourdough fermented wheat germ (SFWG) and raw wheat germ (RWG) incubated at 30°C for 72 h. SFWG was started with *Lactobacillus plantarum* LB1 and *Lactobacillus rossiae* LB5 at the cell density of ca. 5 x 10^7^ cfu/g). RWG was incubated without bacterial inoculum and used as the control. Data are the mean of three independent experiments. Bars of standard deviations are represented.

Aiming at describing the kinetic of release, aliquots of SFWG were analyzed at different intervals of time during 72 h of incubation at 30°C. Further incubation of RWG (control), did not allow a significant (*P* > 0.05) increase of the concentration of 2-methoxy benzoquinone and 2,6-dimethoxybenzoquinone (Figure 2). Nevertheless, a significant (*P* < 0.05) decrease of the value of pH was found (pH 5.02 ± 0.13) under these conditions. A first significant (*P* < 0.05) increase of the concentration of 2-methoxy benzoquinone and 2,6-dimethoxybenzoquinone which corresponded to 102 and 138%, respectively, was already found after 2 h of incubation of SFWG (Figure 2). The concentrations of the two quinones further increased until 24 h, observing the highest increment between 12 and 18 h of incubation. After 48 h of incubation, SFWG contained 2-methoxy benzoquinone and 2,6-dimethoxybenzoquinone at the levels of 0.415 ± 0.016 and 0.252 ± 0.013 mg/g, respectively. Nevertheless, these small increases corresponded to a decay of the organoleptic characteristics of the wheat germ (data not shown), that become too acidic to taste. Not significant (*P* > 0.05) increases of the concentrations of the two quinones were found during late incubation.

### Anti-proliferative activity of SFWG in human cancer cell lines

**Figure 3:**
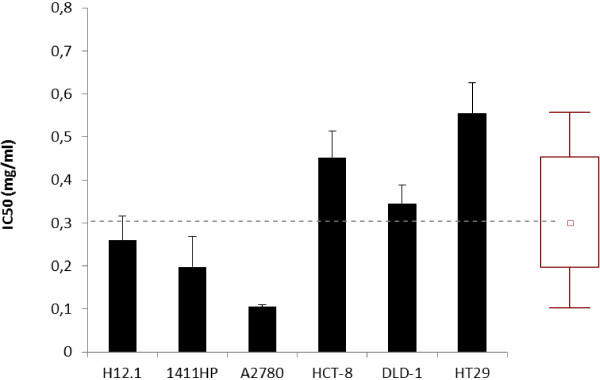
**Anti-proliferative activity on human cell lines.** Anti-proliferative activity of during 96 h of continuous exposure to sourdough fermented wheat germ (SFWG), as determined on different human tumor cell lines (germ cell tumor H12.1, 1411HP; colon carcinoma HCT-8, HT-29, DLD-1; ovarian carcinoma, A2780) using the SRB assay. IC_50_-values are the means of three independent experiments twice analyzed, and related bars of standard deviations are represented. The aggregate data are shown in a box plot. The center line of the box represents the median (□), the top and bottom of the box represent the 25th and 75th percentile of the data, respectively. The top and bottom of the bars represent the 5th and 95th percentile of the data, respectively.

The anti-proliferative activity during 96 h of continuous exposure to SFWG was determined on different human tumor cell lines, using the SRB assay. The values of IC_50_ were calculated using Sigma Plot and the collected data are summarized in Figure 3. The values of IC_50_ of SFWG ranged from 0.105 ± 0.005 to 0.556 ± 0.071 mg/ml, with a median value of IC_50_ of 0.302 mg/ml. The highest anti-proliferative activity was found on ovarian carcinoma cells A2780, while the lowest effect was observed on colorectal carcinoma cells HT-29, followed by HCT-8. The values of IC_50_ for germ cell tumor cells 1411HP and H12.1, and colorectal carcinoma cells DLD-1 were intermediate (0.198 ± 0.071 to 0.344 ± 0.044 mg/ml). No anti-proliferative activity was found for RWG in the range of concentration used.

## Discussion

Quinones consist of a class of bioactive compounds with promising potential as components for anticancer chemoteraphy drugs [11]. Concerning vegetable foods, wheat germ is probably the best reservoir of the glycosylated and non-active forms of 2-methoxy benzoquinone and 2,6-dimethoxybenzoquinone. The non-glycosylated forms of both these compounds have antimicrobial and immune-stimulatory effects [12,13]. In particular, 2,6-dimethoxy-1,4-benzoquinone, which was identified in a large variety of plant families in addition to wheat [14-16], exerts a strong *in vitro* cytotoxicity against human tumor cell lines [13,17,18].

The conversion of glycosylated to non-glycosylated forms requires β-glucosidase activity, which allows a remarkable increase of the functional activities, including the proven anticancer effect [12]. The use of *Lactobacillus zeae* and *Pichia pijperi* as starters for wheat germ fermentation promoted the release of the non-glycosylated forms of 2-methoxy benzoquinone and 2,6-dimethoxybenzoquinone during 48 h of incubation [12].

A fermented and well standardized extract of wheat germ (trade-name Avemar®) was extensively studied for treatment of cancer and autoimmune diseases [19-21]. Avemar® is made through fermentation of wheat germ with baker’s yeast (*Saccharomyces cerevisiae*), which harbors β-glucosidase activity. The aglycone-type 2-methoxy benzoquinone and 2,6-dimethoxybenzoquinone were recognized as the most important bioactive compounds of this preparation [13,21]. The protocol for the manufacture of Avemar® includes the use of a water-soluble extract of wheat germ, the fermentation of this extract, and further concentration and drying. Avemar® induced apoptosis in many cancer cell types, including leukemia, melanoma, breast, colon testicular, head and neck, cervical, ovarian, gastric, thyroid, and brain carcinomas [7,22,23]. The active compounds contained in the fermented wheat germ interfere with anaerobic glycolysis, pentose cycle, and ribonucleotide reductase. Tumor cells were killed by the induction of apoptosis via the caspase-poly [ADP-ribose] polymerase-pathway, interacting with different anticancer drugs [8].

Like *S. cerevisiae*, lactic acid bacteria are food-grade microorganisms largely used for the manufacture of a variety of fermented foods. β-Glucosidase activity is widespread within lactic acid bacteria, even though the level of expression is dependent on the strain [24]. β-Glucosidase activity of lactic acid bacteria was already successfully used to release equol and bioactive isoflavone aglycones from soymilk [24,25].

Lactic acid bacteria isolated from wheat germ were characterized and selected based on technology performance to be used as starters for fermenting wheat germ [1]. Fermentation with selected and autochthonous lactic acid bacteria allowed a long storage of wheat germ, mainly due to lipase inactivation [1,9]. Besides, the use of the sourdough fermented wheat germ as an ingredient for the manufacture of white bread, allowed a noticeable improvement of the nutritional, texture and sensory characteristics [9].

The biothechnology protocol, which was previously used for sourdough fermentation of wheat germ [1], was used in this study to exploit the potential of releasing the non-glycosylated forms of the bioactive compounds 2-methoxy benzoquinone and 2,6-dimethoxybenzoquinone. First, a large *in vitro* screening was carried out based on β-glucosidase activity. Although this enzyme activity was rather diffuse within lactic acid bacteria isolated from wheat germ [1], *L. plantarum* LB1 and *L. rossiae* LB5 were chosen mainly for the potential advantages related to the use of the fermented wheat germ as a food ingredient or dietary supplement and used in combination [1,9]. Overall, both the strains showed very high β-glucosidase activity compared to other lactic acid bacteria isolated from other food ecosystems [24,26], although the main contribution to the release of the two benzoquinones is due to *L. plantarum* LB1. Indeed, the release of the bioactive benzoquinones seems almost proportional to the β-glucosidase activity, and only a slight increase (ca. 30%) was found when *L. rossiae* LB5 was co-inoculated with *L. plantarum* LB1.

It was previously observed that many autochthonous lactic acid bacteria selected for technological properties are at the same time characterized by high β-glucosidase activity, since hypothesizing a role of the enzyme in specific adaptation to cereal [27,28] or similar [29] matrices containing β-glycosides. Moreover, it was already reported that many *L. plantarum* strains possess β-glucosidase activity higher than strains belonging to other lactic acid bacteria species [27-29].

As expected, the sourdough fermented wheat germ was characterized by a low lipase activity compared to the raw matrix, thus ensuring the stability of the preparation to rancidity. Moreover, it showed microbiological, physico-chemical, and biochemical features which mirrored those reported in previous studies [1,9,10]. In agreement with the β-glucosidase assay, the non-glycosylated forms of 2-methoxy benzoquinone and 2,6-dimethoxybenzoquinone were abundantly released during fermentation. An increase of ca. 4 and 6-folds was found compared to levels that were present in the raw wheat germ. The release of the bioactive compounds was almost completed within 24 h and the incubation of the raw wheat germ, without bacterial inoculum under the same conditions did not allow any increase of the non-glycosylated forms. No data regarding the identification and the concentration of the precursors of the bioactive benzoquinones in wheat germ were found in literature, therefore is not possible to confirm the complete conversion of the glycosylated compounds. Raw wheat germ, incubated in the same conditions, did not show detectable increases of the bioactive benzoquinones concentration, probably as the consequence of the marked lower density of lactic acid bacteria and the abundance of strains with low or moderate β-glucosidase activity.

The cytotoxic activity of the sourdough fermented wheat germ was assayed towards several human tumor cell lines to determine potential antitumor features. Usually, human tumor cell lines serve as models for preclinical drug screening [7]. As shown by the values of IC_50_, a significant anti-proliferative activity was found for the sourdough fermented wheat germ. The values were in the range of those previously reported for the preparation Avemar® [7]. Similarly to what found for Avemar®, also the sourdough fermented wheat germ had a narrow range of IC_50_ values and showed a potential clinical activity towards a large spectrum of tumor entities used in the cell line screen. The highest activity was found against A2780 ovarian cancer cells.

## Conclusions

Up to 89% of patients with cancer use integrative, complementary or alternative therapies, which often include herbal or natural products [30]. Natural, nontoxic regimens that enhance standard-of-care therapy and/or prolong progression-free survival, while maintaining the quality of life care are highly desirable in the treatment of cancer [30]. The fermented wheat germ extract Avemar® belongs to the group of nutraceuticals, which are approved as dietary foods for special medical purposes. At the recommended doses, this preparation is well tolerated and possesses a broad therapeutic window [31]. No meaningful toxicity, mutagenicity or genotoxicity were found in preclinical and clinical trials, and no reduction of the activity of conventional chemotherapy was observed [8].

This study demonstrated that selected lactic acid bacteria fermentation of wheat germ released the non-glycosylated and bioactive forms of 2-methoxy benzoquinone and 2,6-dimethoxybenzoquinone, and represent a comparable alternative to *S. cerevisiae* used in the Avemar® preparation. Besides, the same lactic acid bacteria fermentation improves the nutritional, organoleptic and technological features of wheat germ [1,9,10], thus widening its use as a food ingredient, nutritional supplement and/or anticancer drug. Nevertheless, the confirmation of the preclinical cytotoxic activity results by the use of other *in vitro* models and clinical trials is required.

## Methods

### Microorganisms

Forty lactic acid bacteria strains, which were previously isolated from wheat germ, and corresponded to the species *Pediococcus pentosaceus* (10 strains), *Weissella confusa* (21), *Lactobacillus plantarum* (8) and *Lactobacillus rossiae* (1), were used in this study. All strains belong to the Culture Collection of the Department of Soil, Plant and Food Science (University of Bari, IT). Lactic acid bacteria were cultivated on modified MRS (mMRS, maltose and fresh yeast extract were added to MRS at 1 and 5%, respectively, and the final pH was 5.6) until the late exponential phase of growth was reached (ca. 10 h), as previously determined by the analysis of the kinetics of growth (data not shown). To be used for sourdough fermentation of wheat germ, cells were harvested by centrifugation (10,000 x *g*, 10 min, 4°C), washed twice in 50 mM phosphate buffer, pH 7.0, and re-suspended in tap water. To assay the β-glucosidase activity, cultures were incubated for 24 h under the above conditions. Mesophilic lactic acid bacteria in samples were enumerated by plate count on mMRS, at 30°C for 48–72 h, under anaerobiosis. Yeasts were plated on Yeast extract-Peptone-Dextrose agar (YPD, Oxoid, Basingstoke, Hampshire, United Kingdom), added of 150 ppm chloramphenicol, at 30°C for 72 h.

### β-glucosidase activity

Twenty-four hours old cultures were harvested by centrifugation (10,000 x *g*, 10 min at 4°C), washed twice with sterile 50 mM potassium phosphate buffer, pH 7.0, and re-suspended in sterile distilled water at the 620 nm absorbance of 2.5, which corresponded to ca. 2 x 10^9^ cfu/ml. These cell suspensions were used to assay β-glucosidase activity. The β-glucosidase activity was measured in terms of *p*-nitrophenol released from *p*-nitrophenyl-β-D-glucopyranoside (*p*NPG) substrate (Sigma) [32]. The assay mixture contained 900 μL of 2.5 mM *p*NPG (final concentration) in 0.5 M potassium phosphate buffer, pH 7.5, and 100 μL of cell suspension. The mixture was incubated at 40°C for 30 min and the reaction was stopped by heating the mixture at 95°C for 5 min. The absorbance was measured at 410 nm. One unit (U) of enzyme activity was defined as the amount of β-glucosidase that released 1 μmol of *p*-nitrophenol from the substrate *p*NPG per milliliter per min under the assay conditions [33]. Calibration curve for *p*-nitrophenol (Sigma) was obtained using concentrations in the range of 0.05-2 mM.

### Wheat germ and sourdough fermentation

Six batches of wheat germ were supplied by the industry Tandoi Pellegrino (Corato, Bari, Italy) and pooled before use. The germ was separated from refined flour during milling of *Triticum aestivum* cv. Appulo by a degerminator and a set of rollermills (Bühler AG, Uzwil, Switzerland). Samples of wheat germ were characterized for moisture, ash and fat according to the Approved Methods 44–16, 08–01 and 30–10 of the American Association of Cereal Chemists, respectively [34]. Proteins were determined by Kjeldahl method. TTA was determined on 10 g of wheat germ homogenized with 90 ml of distilled water and expressed as the amount (ml) of 0.1 M NaOH to get pH of 8.3. The values of pH were determined by a Foodtrode electrode (Hamilton, Bonaduz, Switzerland).

Water/salt-soluble extracts from raw or sourdough fermented wheat germ were prepared according to the method originally described by Osborne [35], and modified by Weiss et al. [36]. Fifteen grams of sample were suspended in 60 ml of 50 mM Tris–HCl (pH 8.8), held at 4°C for 1 h, vortexing at 15 min intervals, and centrifuged at 20,000 *x g* for 20 min. The supernatant was used for analyses. Organic acids from the water/salt-soluble extract were determined by HPLC (High Performance Liquid Chromatography), using an ÄKTA Purifier system (GE Healthcare), equipped with an Aminex HPX-87H column (ion exclusion, Biorad) and a UV detector operating at 210 nm. Elution was at 60°C, with a flow rate of 0.6 ml/min, using H_2_SO_4_ 10 mM as mobile phase [37]. Total and individual free amino acids of the water/salt-soluble extract were analyzed by a Biochrom 30 series Amino Acid Analyzer (Biochrom Ltd., Cambridge Science Park, England) with a Na-cation-exchange column (20 by 0.46 cm inner diameter) as described by Rizzello et al. [38]. Organic acids and amino acids used as standards were purchased from Sigma Chemical Co. (Milan, Italy).

Preliminarily, *L. plantarum* LB1, *L. rossiae* LB5, previously selected on the basis of technological properties [1,9] and for the high β-glucosidase activity, and *W. confusa* G8, and *P. pentosaceus* G1, respectively representative of the strains having β-glucosidase activity values close to the median or of the strains having very low enzymatic activity, were used (singly) to ferment wheat germ, aiming at verifying the different release of the benzoquinones. Lactic acid bacteria were used at the final cell density of ca. 5 x 10^7^ cfu/g of dough (dough yield DY, dough weight x 100/flour weight, 160), and fermentation was allowed at 30°C for 24 h. All further analyses were carried out on sourdough fermented wheat germ (SFWG) inoculated with *L. plantarum* LB1 and *L. rossiae* LB5 together, as previously described [1,9].

Aiming at describing the kinetic of release of quinones, the fermentation of wheat germ was also allowed for 72 h, collecting samples at 2, 4, 8, 12, 18, 24, 48, and 72 h. SFWG was freeze dried, and used for analyses. Wheat germ, without bacterial inoculum, was prepared, incubated under the same conditions, and used as the control. Raw and fermented wheat germ samples were analyzed at least in triplicate.

Serial dilution of freeze dried SFWG were made and plated onto mMRS (Oxoid LTD). Enumeration of lactic acid bacteria was carried out at 30°C for 48 h. In order to differentiate the colonies of the two strains, DNA was extracted from colonies of the highest plate dilutions of mMRS and used for RAPD-PCR analysis, as described by Minervini et al. [39]. RAPD-PCR analysis was carried out as reported by Coda et al. [40], using primers P7 (5’ AGCAGCGTGG 3’) and M13 (5’-GAGGGTGGCGGTTCT-3’) (Invitrogen, Milan, Italy).

### Lipase activity

Water/salt-soluble extracts from raw wheat germ and sourdough fermented wheat germ were prepared according to the method originally described by Osborne and modified by Weiss et al. [36]. As previously shown [41], wheat germ lipase has good solubility in water/salt-buffers. The concentration of proteins in the water/salt-soluble extracts was determined by the Bradford method [42]. Tributyrin as the substrate and the agar diffusion assay [43] were used to determine the lipase activity of the water/salt-soluble extracts. Agar plates contained 1% (w/v) of triglyceride, 0.02% (wt/vol) sodium azide, and 50 mM phosphate buffer, pH 8.0. As reported by Kapranchikov et al. [44], this value of pH was the optimum for wheat germ endogenous lipase activity. Lipase activity was expressed as the minimum dilution of the enzyme preparation that failed to give a detectable zone of hydrolysis after 24 h of incubation at 30°C.

### Extraction of 2-methoxy benzoquinone and 2,6-dimethoxybenzoquinone

Raw and sourdough fermented wheat germ were separately freeze dried and milled. Ten grams of each sample were dissolved into 250 ml of double distilled water and subjected three times to extraction by shaking with 100 ml of chloroform (CHCl_3_) [18]. CHCl_3_ layers were pooled, washed twice with distilled water, and dried over anhydrous Na_2_SO_4_. The extract was evaporated to dryness under a vacuum evaporation at 35°C. The dry material was re-dissolved in CHCl_3_ and filtered through a 0.22 μm PTFE filter aid. Twenty microliters of the filtrate were analyzed by HPLC.

### High-pressure liquid chromatography analysis

The concentration of 2-methoxy benzoquinone and 2,6-dimethoxybenzoquinone of wheat germ extracts was determined by High-Pressure Liquid Cromatography (HPLC), using the method of Tömösközi-Farkas, and Daood [13] with some modifications. The HPLC system consisted of an Äkta purifier HPLC, equipped with a XTerraTM MS C18 5 μm, 4.6x250 mm column (Waters, Brussels, Belgium) and a detector UV900 (GE Healthcare Bio-Sciences Corp., Piscataway, New Jersey, USA) operating at 275 nm. The mobile phase was a water:acetonitrile mixture [80:20 (v/v)], containing 0.0025 M KH_2_PO_4_, with flow rate and sample injection volume fixed at 0.7 ml/min and 20 μl, respectively. 2-Methoxy benzoquinone and 2,6-dimethoxybenzoquinone (Sigma) dissolved in 100% CHCl_3_ were used as the references to calibrate the standard curve and retention times.

### Cell lines and cultures

The following human cancer cell lines, which belong to the Collection of the Department of Internal Medicine IV, Oncology/Hematology, Martin-Luther-University Halle-Wittenberg, Halle/Saale, Germany), were used for assays: germ cell tumor (H12.1, 1411HP), colon carcinoma (HCT-8, HT-29, DLD-1) and ovarian carcinoma (A2780). All cell lines were grown as monolayers up to 80% of confluence in RPMI1640, supplemented with 10% fetal bovine serum (FBS) and 1% Penicillin/Streptomycin, at 37°C, in the presence of 5% CO_2_ and humidified air.

### Growth inhibition assays

The anti-proliferative activity was assayed using the total protein sulforhodamine B (SRB) method [45]. Briefly, cells were seeded into 96-well plates at a cell line specific density (ca. 2 x 10^4^ cells/well) to ensure exponential growth throughout the whole period of the assay. The optimal cell numbers was previously set up by determining growth kinetics [7]. After 24 h, exponentially growing cells were exposed to serial dilutions (0.05 - 2.00 mg/ml) of raw and sourdough fermented wheat germ for 96 h. After assay, media were removed, cells were fixed with 10% TCA and processed according to the SRB protocol proposed by Skehan et al. [45]. Absorbance was measured at 570 nm, using a 96-well plate reader (Rainbow, SLT, Germany). Dose response curves were generated by Sigma Plot (Janded Scientific, San Rafael CA) and IC_50_ values were calculated based on the Hill equation [7].

### Statistical analysis

Data were subjected to one-way ANOVA; pair-comparison of treatment means was achieved by Tukey’s procedure at *P*<0.05, using the statistical software Statistica 8.0 (StatSoft Inc., Tulsa, USA).

## Competing interest

The authors declare that they have no competing interest.

## Authors’ contributions

CGR carried out purification and identification of bioactive compounds and experimental design of the work; RC carried out microbiological and chemical analyses, and elaboration of the data; LN and JAC performed fermentations and microbiological determination; TM, and FR carried out ex-vivo assays and related experimental procedures; MG was the supervisors and the coordinators of the research units. All authors read and approved the final manuscript.
